# A Novel Radiographic Pattern Related to Poor Prognosis in Patients with Prostate Cancer with Metastatic Spinal Cord Compression

**DOI:** 10.1016/j.euros.2022.12.004

**Published:** 2022-12-26

**Authors:** Johan Wänman, Kasim Abul-Kasim, Julius Semenas, Elin Thysell, Anders Bergh, Pernilla Wikström, Sead Crnalic

**Affiliations:** a Department of Surgical and Perioperative Sciences, Orthopaedics, Umeå University, Umeå, Sweden; b Division of Neuroradiology, Diagnostic Centre for Imaging and Functional Medicine, Faculty of Medicine, Lund University, Skåne University Hospital, Lund, Sweden; c Department of Medical Biosciences, Pathology, Umeå University, Umeå, Sweden

**Keywords:** Metastatic spinal cord compression, Myeloma-like prostate cancer bone metastases, Prostate cancer

## Abstract

**Background:**

Prostate cancer spinal bone metastases can have a radiographic profile that mimics multiple myeloma.

**Objective:**

To analyse the presence and prognostic value of myeloma-like prostate cancer bone metastases and its relation to known clinical, molecular, and morphological prognostic markers.

**Design, setting, and participants:**

A cohort of 110 patients with prostate cancer who underwent surgery for metastatic spinal cord compression (MSCC) was analysed. Spinal bone metastases were classified as myeloma like (*n* = 20) or non–myeloma like (*n* = 90) based on magnetic resonance imaging prior to surgery. An immunohistochemical analysis of metastasis samples was performed to assess tumour cell proliferation (percentage of Ki67-positive cells) and the expression levels of prostate-specific antigen (PSA) and androgen receptor (AR). The metastasis subtypes MetA, MetB, and MetC were determined from transcriptomic profiling.

**Outcome measurements and statistical analysis:**

Survival curves were compared with the log-rank test. Univariate and multivariate Cox proportional hazard models were used to assess the effects of prognostic variables. Groups were compared using the Mann-Whitney *U* test for continuous variables and the chi-square test for categorical variables.

**Results and limitations:**

Patients with the myeloma-like metastatic pattern had median survival after surgery for MSCC of 1.7 (range 0.1–33) mo, while the median survival period of those with the non–myeloma-like pattern was 13 (range 0–140) mo (*p* < 0.001). The myeloma-like appearance had an independent prognostic value for the risk of death after MSCC surgery (adjusted hazard ratio 2.4, *p* = 0.012). Postoperative neurological function was significantly reduced in the myeloma-like group. No association was found between the myeloma-like pattern and morphological markers of known relevance for this patient group: the transcriptomic subtypes MetA, MetB, and MetC; tumour cell proliferation; and AR and PSA expression.

**Conclusions:**

A myeloma-like metastatic pattern identifies an important subtype of metastatic prostate cancer associated with poor survival and neurological outcomes after surgery for MSCC.

**Patient summary:**

This study describes a novel radiographic pattern of prostate cancer bone metastases and its relation to poor patient prognosis.

## Take Home Message

We found a novel radiographic pattern of prostate cancer bone metastases on magnetic resonance imaging that was similar to haematological malignancies such as multiple myeloma. This pattern was associated with poor survival and neurological outcomes after surgery for metastatic spinal cord compression.

## 1 Introduction

Prostate cancer is the most common malignant tumour in men [1]. Most patients with advanced prostate cancer develop bone metastases, and the spine is the most common metastatic site [2]. Progressive destruction of the bone in the affected vertebrae may cause pathological fractures and metastatic spinal cord compression (MSCC), with para/tetra paresis and incontinence, severely reducing patient quality of life and negatively impacting survival outcomes.

Prostate cancer bone metastases are generally classified as osteoblastic [3]. There is, however, an overlap between osteolytic and osteoblastic activity in bone metastases, and the categorisation is likely oversimplified. Bone resorption markers have been reported to be particularly elevated in prostate cancer bone metastases compared with those from other malignancies [4], [5], and osteolytic bone-related parameters have been shown to be a negative prognostic factor for overall survival outcomes in patients with castration-resistant prostate cancer (CRPC) [6]. Prostate cancer bone metastases can have a radiographic profile that mimics multiple myelomas [7]. Presentation with myeloma-like spinal bone metastasis from prostate cancer is rare, with only a few case reports presented in the literature [7], [8], [9]. The myeloma-like subtype of prostate cancer bone metastases has previously been suggested to be osteolytic [7], but little is known about its prognostic value.

Prostate cancer bone metastases are heterogeneous at the genomic, transcriptomic, proteomic, metabolic, and morphological levels [10], [11], [12], [13]. We recently identified three molecular subtypes of prostate cancer bone metastases, named MetA, MetB, and MetC, in a set of clinical bone metastasis samples based on differential gene expression and an unsupervised cluster analysis [14]. The prognostic value and biological relevance of the MetA, MetB, and MetC subtypes have been verified in several independent patient cohorts [15]. The MetA subtype is the most common; it shows high androgen receptor (AR) activity, and MetA patients have a relatively favourable prognosis compared with patients with other subtypes. The MetB subtype has high cell cycle activity and low AR activity, and MetB patients have the worst prognosis. Additionally, the MetC subtype shows low AR activity, in combination with signs of epithelial-to-mesenchymal transition, myogenesis, and an inflammatory response. The MetA and MetB subtypes can be differentiated by an immunohistochemical analysis combining assessments of prostate-specific antigen (PSA; marker for cell differentiation) and Ki-67 (marker for cell proliferation) [14], two markers that, together with AR immunoreactivity (IR), have previously been associated with prognosis in prostate cancer patients with MSCC [10], [13], [14].

The aim of the current study was to analyse the presence and prognostic value of myeloma-like prostate cancer bone metastases in a cohort of 110 patients who underwent surgery for MSCC. We then analysed whether the myeloma-like subtype was related to clinical, molecular, and morphological markers previously reported to be of prognostic relevance in prostate cancer patients with bone metastases, and furthermore, whether these metastases had a more osteolytic pattern based on computed tomography (CT) and magnetic resonance (MR) imaging (MRI), and immunohistochemistry.

## 2 Patients and methods

### 2.1 Patients

The study material was obtained from a retrospective analysis of 110 consecutive patients with MSCC due to prostate cancer who underwent surgery at the Department of Orthopaedics, Umeå University Hospital, between 2003 and 2017. The cohort has been described previously [16]. Tissue samples from bone metastases were collected during surgery and stored as freshly frozen or formalin-fixed, paraffin-embedded samples, as described previously [12], [17]. The diagnosis of prostate cancer metastasis was confirmed histologically. The follow-up time was defined as the time between primary tumour diagnosis, the time of the start of androgen deprivation therapy (ADT) or surgery for MSCC, and the date of the latest follow-up (May 1, 2021) or death. The study is a part of a wider research project on prostate cancer bone metastasis that was approved by the regional ethical review board of Umeå University (Dnr: 223/03, 03-185, 04-26M [August 24, 2007], 03-158).

### 2.2 Radiographic classification

Identification of the radiographic features of spinal bone metastases and grading of the Spinal Instability Neoplastic Score [18] and the Epidural Spinal Cord Compression scale [19] were based on preoperative MR images and CT scans, which were analysed by a neuroradiologist (K.A.-K.) who was blinded to the preoperative clinical data and outcomes.

### 2.3 Morphological analysis

The bone volume density in the sections was determined by mounting a square lattice on the eyepiece of a light microscope, and counting the fraction of grid intersections on the bone and other components of the metastatic tissue.

An immunohistochemical analysis of Ki67, PSA, and AR levels was performed and evaluated as described previously [10]. In brief, PSA and AR IR was assessed using a scoring system based on the percentage (0 = no staining, 1 = 1–25%, 2 = 26–50%, 3 = 51–75%, and 4 = 76–100%) and intensity (0 = no staining, 1 = weak, 2 = moderate, and 3 = intense) of tumour epithelial cell staining. An IR score was obtained by multiplying the scores for distribution and intensity, as described previously [10], resulting in IR scores ranging from 0 to 12. The tumour cell proliferation index was assessed as the percentage of tumour epithelial cells showing positive Ki67 staining [10]. The MetA, MetB, and MetC subtypes were determined from transcriptomic profiles (GSE29650 and GSE101607), as described previously [14].

### 2.4 Statistical analysis

Descriptive statistics of continuous variables were expressed as medians (ranges), while categorical data were expressed as numbers and percentages. Groups were compared using the Mann-Whitney *U* test for continuous variables, and the chi-square test or Fisher’s exact test for categorical variables. Survival was estimated by a Kaplan-Meier survival analysis with death from prostate cancer as an event. Survival curves were compared with the log-rank test. Univariate and multivariate Cox proportional hazard models were used to assess the effects of prognostic variables. The results were expressed as hazard ratios with corresponding 95% confidence intervals. A *p* value of <0.05 was considered statistically significant. Statistical analysis was performed using IBM SPSS Statistics, version 25 (IBM Corporation, Armonk, NY, USA).

## 3 Results

The clinical characteristics and treatment prior to surgery are summarised in Table 1. The proportion of patients with lower Karnofsky performance status (KPS <80) was higher in the myeloma-like group than in the non–myeloma-like group prior to surgery for MSCC (*p* = 0.026), while no other statistically significant differences were observed between the groups (Table 1). Adjuvant radiation therapy was given to eight patients in the myeloma-like group at a median dose of 28 (24–28) Gy and at a median interval of 31 (23–41) d postoperatively. Postoperative adjuvant radiation was given to 54 patients in the non–myeloma-like group at a median dose of 24 (16–28) Gy and a median interval of 36 (23–125) d.

**Table 1:** Clinical characteristics of the patients who underwent surgery for metastatic spinal cord compression (MSCC) a

Variables	Myeloma-like prostate cancer bone metastases (*n* = 20)	Non–myeloma-like prostate cancer bone metastases (*n* = 90)	*p* value
Age at surgery for MSCC	70 (61–88)	74 (50–88)	0.063
Preoperative KPS b			0.026
80–100%	6 (30)	53 (59)	
50–70%	14 (70%)	37 (41)	
PSA at diagnosis of primary tumour c	140 (0–5000)	99 (0–10000)	0.56
PSA at surgery for MSCC	300 (13–5000)	140 (0.06–10000)	0.19
Gleason score of primary tumour			0.56
≤6	1 (5)	5 (6)	
7	9 (45)	23 (26)	
8–10	6 (30)	29 (32)	
Not available	4 (20)	33 (37)	
Hormone status at MSCC diagnosis			0.15
Hormone naïve	2 (10)	24 (27)	
Castration resistant	18 (90)	66 (73)	
Bone metastases present at diagnosis of primary tumour	14 (70)	57 (63)	0.62
Treatment of prostate cancer before surgery for MSCC			
Radical prostatectomy	2 (10)	2 (2)	0.15
Curative radiotherapy	3 (15)	7 (8)	0.39
ADT			0.31
Orchidectomy	4 (20)	17 (19)	
GnRH	15 (75)	56 (62)	
Chemotherapy	5 (25)	20 (22)	0.78
Functional status prior to surgery			
Ambulatory	3 (15)	22 (24)	0.56
Nonambulatory	17 (85)	68 (76)	

ADT = androgen deprivation therapy; GnRH = Gonadotropin-releasing hormone; KPS = Karnofsky performance status; PSA = prostate-specific antigen.

a Data are presented as the median (range) or *n* (%).

b Karnofsky performance status scale: 100—normal, no complaints, no evidence of disease; 90—able to carry on normal activity, minor signs of symptoms of disease; 80—normal activity with effort, some signs or symptoms of disease; 70—cares for self, unable to carry on normal activity or to do any work; 60—requires occasional assistance from others but able to care for most of their own needs; 50—requires considerable assistance from others and frequent medical care; 40—disabled, requires special care and assistance; 30—severely disabled, hospitalisation indicated, death not imminent; 20—very sick, hospitalisation necessary, active supportive treatment necessary; 10—moribund; and 0—dead. There were no patients with a KPS score of <50%.

c PSA was not available at the time of surgery for one patient in the myeloma-like group and 13 in the non–myeloma-like group.

### 3.1 Radiographic pattern of prostate cancer bone metastases

**Fig. 1:**
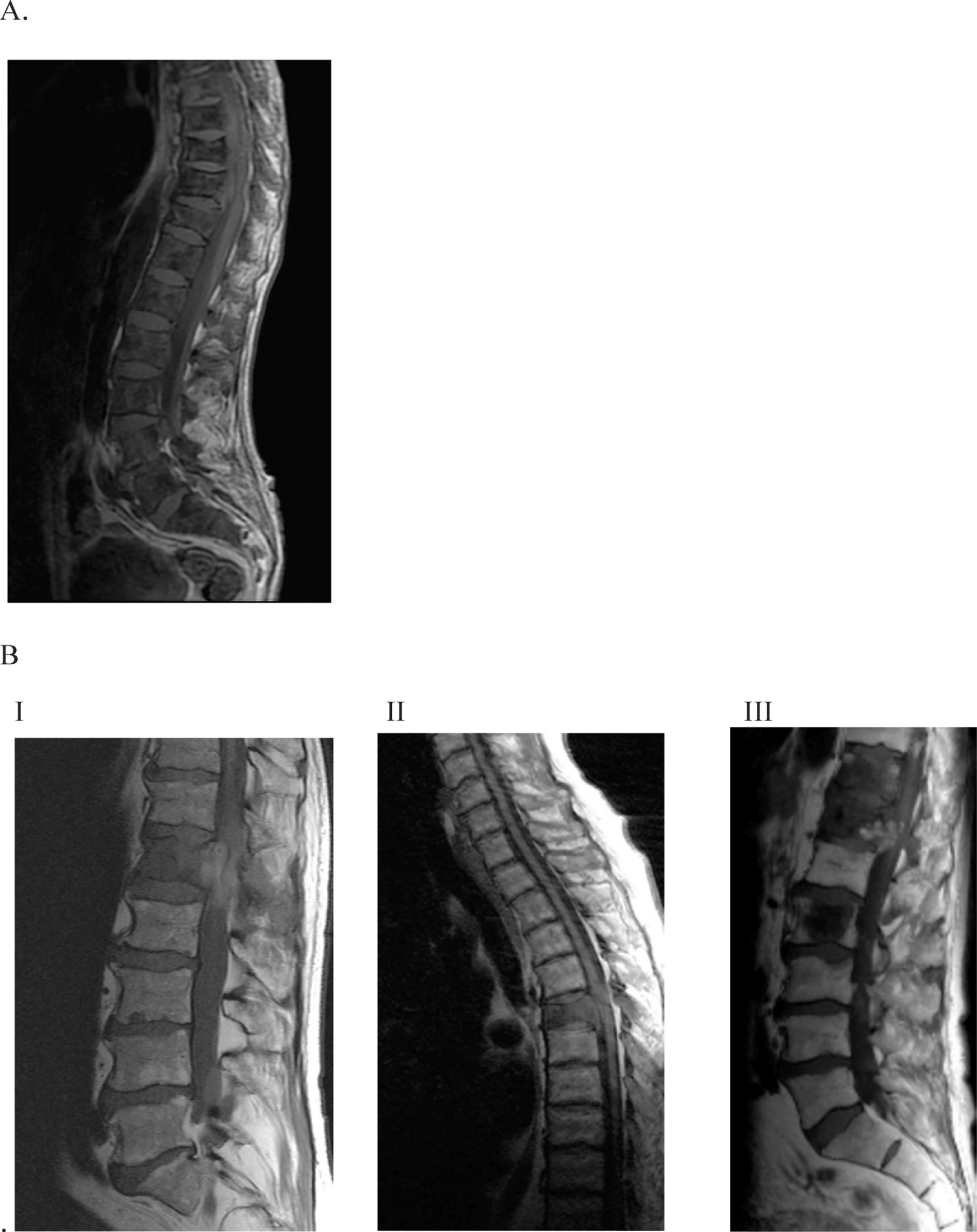
T1-weighted MR images of the subtypes of MSCC from prostate cancer: (A) myeloma-like metastases with diffuse infiltrating that replace the normal fatty bone marrow; (B) non–myeloma-like bone metastases: I, single metastatic lesion causing MSCC; II, multiple small metastases, of which one large metastasis causes MSCC; and III, multiple small and large metastases, of which one large metastasis causes MSCC. MR = magnetic resonance; MSCC = metastatic spinal cord compression.

The pattern of the metastatic spread and distribution in the vertebral column with diffuse infiltrating metastases that replaced the normal fatty bone marrow (a pattern similar to myelomas and other haematological malignancies such as leukaemia or lymphoma) was used to categorise prostate cancer bone metastases as myeloma like (*n* = 20) or non–myeloma like (*n* = 90). The myeloma-like appearance on the MRI showed a low signal on T1-weighted images (Fig. 1A) and a high signal on T2-weighted images. In the non–myeloma-like group, the following three different modes of metastatic spread were observed: a single metastatic lesion causing MSCC (*n* = 9), multiple small metastases with one large metastasis causing MSCC (*n* = 14), and multiple small and large metastases with one large metastasis causing MSCC (*n* = 67; Fig. 1B). The other radiographic parameters were similar in the myeloma- and non–myeloma-like groups (Table 2).

**Table 2:** Radiographic features at the time of surgery for metastatic spinal cord compression (MSCC) a

	Myeloma-like prostate cancer bone metastases (*n* = 20)	Non–myeloma-like prostate cancer bone metastases (*n* = 90)	*p* value
Site of MSCC			0.77
Cervical	0	1 (1)	
Cervical + thoracic	0	2 (2)	
Thoracic	15 (75)	71 (79)	
Lumbar	5 (25)	16 (18)	
SINS			0.87
Stable	1 (5)	3 (3)	
Potentially unstable	17 (85)	75 (83)	
Unstable	2 (10)	12 (13)	
ESCC scale			0.33
1	3 (15)	23 (26)	
2	9 (45)	26 (29)	
3	8 (40)	41 (46)	
Total tumour infiltration in the vertebrae at the site of MSCC	18 (90)	57 (63)	0.05
Radiographic signs of myelopathy	9 (45)	46 (51)	0.65
Spinal cord oedema	4 (20)	26 (29)	0.63
Appearance at the site of MSCC			
Osteolytic or mixed (osteoblastic and osteolytic)	19 (95)	87 (97)	0.56
Pure osteoblastic	1 (5)	3 (3)	

ESCC = Epidural Spinal Cord Compression scale [19]; SINS = Spinal Instability Neoplastic Score [18].

a Data are presented as *n* (%).

### 3.2 Survival

**Fig. 2:**
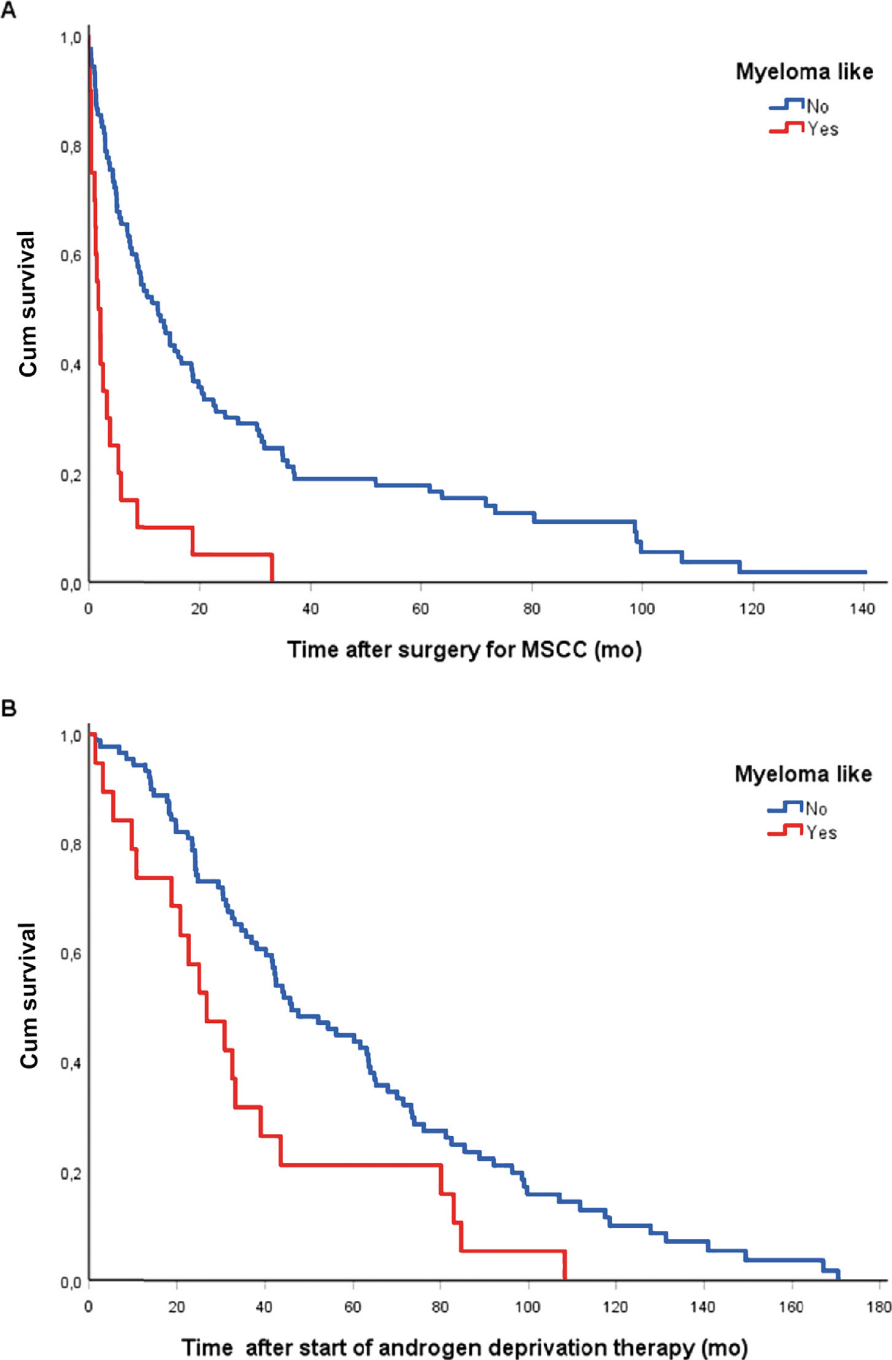

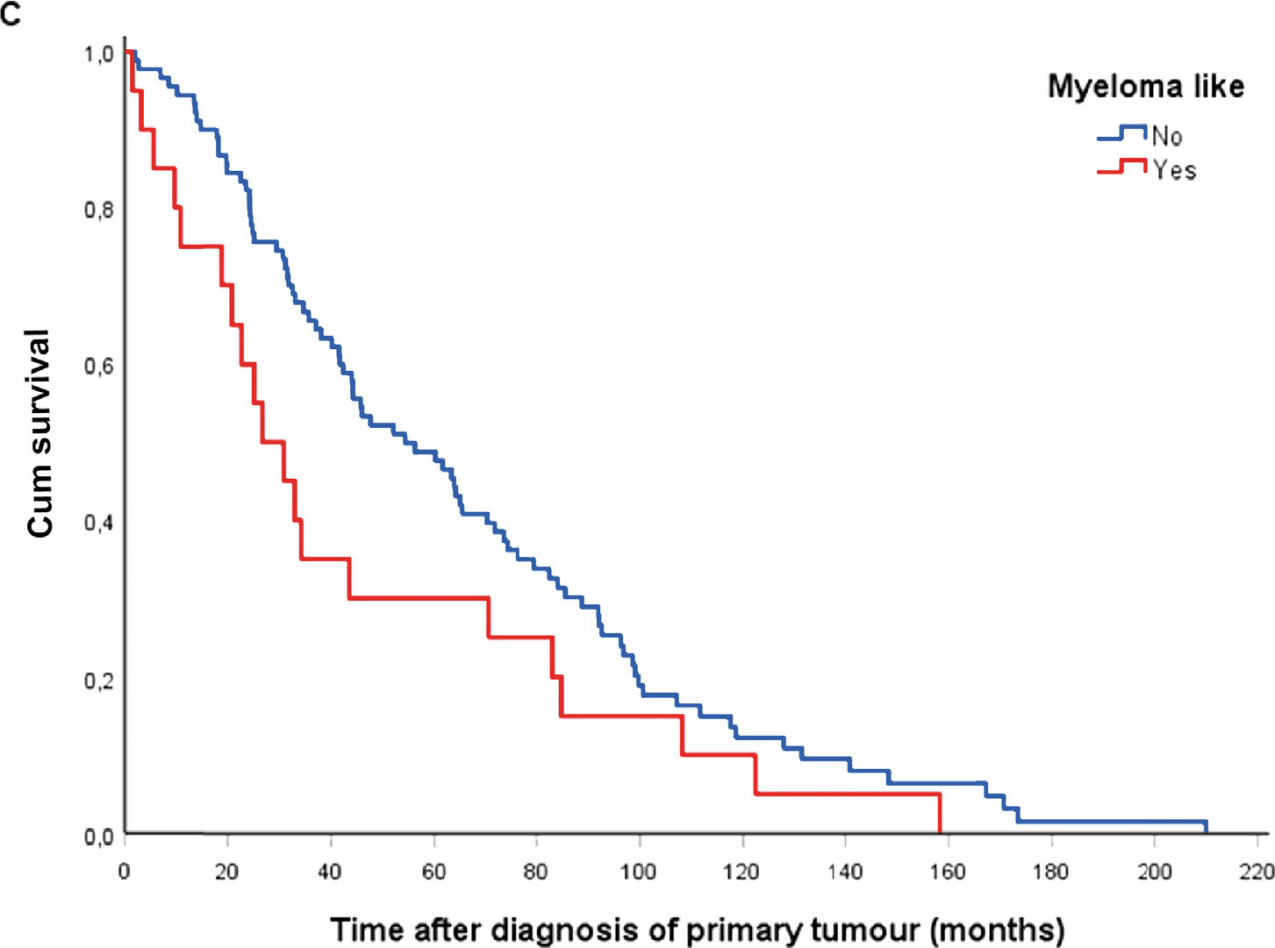
Survival analysis of the patients with myeloma-like and non–myeloma-like prostate cancer bone metastases after (A) surgery for MSCC (*p* < 0.001), (B) the start of androgen deprivation therapy (*p* = 0.01), and (C) the time from primary tumour diagnosis (*p* = 0.07). Cum = cumulative; MSCC = metastatic spinal cord compression.

Patients with the myeloma-like metastatic pattern had a median postoperative survival duration of 1.7 (0.1–33) mo, whereas patients with non–myeloma-like metastases had a median survival time of 13 (0–140) mo (*p* < 0.001; Fig. 2A). The median survival period after the start of ADT was 27 (1.5–108) mo among patients in the myeloma-like group and 46 (1.7–171) mo among those in the non–myeloma-like group (*p* = 0.01; Fig. 2B). The median survival time after diagnosis of primary prostate cancer was 27 (1.5–158) and 54 (2–210) mo for patients with the myeloma-like and the non–myeloma-like metastatic pattern, respectively (*p* = 0.074; Fig. 2C).

The prognostic value of the myeloma-like metastatic pattern was compared with the following markers previously reported to be of prognostic relevance in this patient cohort: the molecular metastasis subtypes MetA, MetB, and MetC; tumour cell proliferation index; PSA and AR IR scores; and KPS [10], [14], [16]. As demonstrated by a multivariate Cox regression analysis, the myeloma-like pattern, PSA IR score, and KPS all provided independent prognostic information for the risk of death after surgery for MSCC (Table 3). When survival after the start of ADT was analysed, however, the PSA IR score was the only marker that provided independent prognostic information, with a higher score associated with a better prognosis (Table 4).

**Table 3:** Univariate and multivariate Cox regression analysis of covariates in relation to mortality after surgery for metastatic spinal cord compression (MSCC)

Variable	Univariate		Multivariate (*n* = 68)	
	HR (95% CI)	*p* value	HR (95% CI)	*p* value
Myeloma like				
No (*n* = 90)	1.0			
Yes (*n* = 20)	3.5 (2.1–5.8)	2E–6	2.5 (1.2–5.1)	0.012
Metastasis subtype				
MetA (*n* = 49)	1.0		1.0	
MetB (*n* = 11)	2.1 (1.0–4.1)	0.038	0.89 (0.35–2.3)	0.81
MetC (*n* = 9)	1.0 (0.49–2.1)	1.0	0.84 (0.39–1.8)	0.64
Proliferation (%) a				
Low (≤25; *n* = 74)	1.0			
High (>25; *n* = 25)	1.8 (1.1–2.9)	0.014	1.3 (0.60–2.8)	0.52
PSA (IR score) b				
Low (≤8; *n* = 60)	1.0			
High (>8; *n* = 41)	0.47 (0.31–0.73)	6.7E-4	0.54 (0.30–0.96)	0.034
AR (IR score) c				
Low (<8; *n* = 38)	1.0			
High (≥8; *n* = 61)	1.5 (0.96–2.2)	0.080		
Age at surgery for MSCC (*n* = 110)	1.0 (0.97–1.0)	0.99		
Serum PSA at surgery for MSCC (*n* = 96) d	1.0 (1.0–1.0)	0.089		
KPS (%)				
Low (50–70; *n* = 51)	1.0			
High (80–100; *n* = 59)	0.40 (0.27–0.61)	1.5E–5	2.6 (1.4–4.8)	0.0017

AR = androgen receptor; CI = confidence interval; HR = hazard ratio; IR score = immunoreactivity score; KPS = Karnofsky performance status; PSA = prostate-specific antigen.

a Samples were available for the proliferation score in 79 patients in the non–myeloma-like group and 20 patients in the myeloma-like group.

b Samples were available for the PSA IR score in 81 patients in the non–myeloma-like group and 20 patients in the myeloma-like group.

c Samples were available for the AR IR score in 81 patients in the non–myeloma-like group and 20 patients in the myeloma-like group.

d PSA was not available at the time of surgery for one patient in the myeloma-like group and 13 patients in the non–myeloma-like group.

**Table 4:** Univariate and multivariate Cox regression analysis of covariates in relation to mortality after the start of androgen deprivation therapy (ADT) for primary prostate cancer

	Univariate		Multivariate	
	HR (95% CI)	*p* value	HR (95% CI)	*p* value
Myeloma like				
No (*n* = 90)	1.0		1.0	
Yes (*n* = 20)	1.9 (1.2–3.2)	0.011	1.7 (0.90–3.2)	0.10
Metastasis subtype				
MetA (*n* = 49)	1.0		1.0	
MetB (*n* = 11)	2.1 (1.0–4.0)	0.037	1.4 (0.71–3.0)	0.31
MetC (*n* = 9)	0.89 (0.46–2.0)	0.95	0.92 (0.44–1.9)	0.82
Proliferation (%) a				
Low (≤25; *n* = 73)	1.0	0.064		
High (>25; *n* = 24)	1.6 (0.98–2.5)			
PSA (IR score) b				
Low (≤8; *n* = 58)	1.0		1.0	
High (>8; *n* = 41)	0.50 (0.32–0.77)	0.0017	0.55 (0.32–0.95)	0.033
AR (IR score) c				
Low (<8; *n* = 37)	1.0			
High (≥8; *n* = 60)	1.0 (0.67–1.6)	0.92		
Age at start of ADT (*n* = 108) d	1.0 (0.99–1.0)	0.26		

AR = androgen receptor; CI = confidence interval; HR = hazard ratio; IR score = immunoreactivity score; PSA = prostate-specific antigen.

a Samples were available for the proliferation score in 79 patients in the non–myeloma-like group and 20 patients in the myeloma-like group.

b Samples were available for the PSA IR score in 81 patients in the non–myeloma-like group and 20 patients in the myeloma-like group.

c Samples were available for the AR IR score in 81 patients in the non–myeloma-like group and 20 patients in the myeloma-like group.

d Two patients were excluded from the ADT analysis due to an uncertain date of ADT therapy.

### 3.3 Neurological outcome

In the myeloma-like group, only three out of the 20 patients could walk prior to surgery. Six of the patients died within 1 mo after surgery, one patient died 6 wk after surgery but was missed at the 1-mo follow-up, and only five of the 13 patients who were alive at 1 mo could walk. In the group with a non–myeloma-like metastatic pattern, 22 of the 90 patients could walk prior to surgery. One month after surgery, 83 patients were alive, of whom 57 were ambulatory, six had died, and one was missed at follow-up. Postoperative ambulatory function was reduced significantly in the myeloma-like group (*p* = 0.034).

### 3.4 Bone metastasis morphology

Markers previously reported to be associated with metastasis aggressiveness in this patient cohort (MetA, MetB, and MetC subtypes; tumour cell proliferation index; and PSA and AR IR scores) were compared between the group with a myeloma-like pattern and the group with a non–myeloma-like pattern (Table 5). As the myeloma-like metastatic pattern had previously been described as potentially osteolytic, we also compared the density of bone within the metastases between the two patient groups. As demonstrated in Table 5, none of the examined markers differed significantly between the two groups.

**Table 5:** Molecular and histopathological characteristics of suggested clinical and biological relevance in metastatic prostate cancer a, b

	Myeloma like	Non–myeloma like	*p* value
Metastasis subtype (*n* = 69)			0.68
MetA	8 (62)	41 (73)	
MetB	3 (23)	8 (14)	
MetC	2 (15)	7 (13)	
Tumour cell proliferation (%)			
*n* = 20 vs 79	19 (3.5–50)	15 (2–80)	0.33
PSA (IR score)			
*n* = 20 vs 81	6 (0–9)	6 (0–12)	0.15
AR (IR score)			
*n* = 20 vs 79	9 (0–12)	8 (0–12)	0.74
Bone density (%)			
*n* = 19 vs 62	5.4 (0–39)	9.2 (0–36)	0.61

AR = androgen receptor; IR score = immunoreactivity score; PSA = prostate-specific antigen.

a Data are presented as the median (range) or *n* (%).

b MetA, MetB, and MetC (metastasis subtypes A, B, and C), tumour cell proliferation, and bone density were defined as described in the methods.

## 4 Discussion

Little is known about the diffuse infiltrative myeloma-like subtype of prostate cancer bone metastases, previously described in only a few case reports [7], [8], [9]. Here, we report this particular radiographic pattern to be present in a relatively high proportion (18%) of the cases operated on for MSCC, and furthermore, to be related to particularly poor survival and reduced ambulatory function after surgery. A morphological analysis did not imply that the myeloma-like phenotype is related to previously known biological risk factors for this patient group, such as tumour cell proliferation, AR activity, bone remodelling, or the recently described metastasis subclasses MetA, MetB, and MetC [14]. Thus, the diffuse infiltrative myeloma-like subtype of prostate cancer bone metastases might be a novel independent predictor of poor prognosis in prostate cancer patients with MSCC and requires further exploration.

MSCC is a serious complication of vertebral metastases that develop due to progressive tumour growth bone destruction and spinal instability. The treatment for MSCC aims to preserve/regain neurological function decrease pain and maintain continence. Spinal surgery in combination with radiotherapy has shown superior outcomes to radiotherapy alone [20] but spinal surgery is associated with considerable morbidity rates [21]. In general surgery is recommended only if the expected survival time exceeds 3–6 mo [22]. The selection of surgical candidates is complex and the identification of prognostic factors for postoperative survival times is important. Prognostic factors for postoperative survival times after spinal surgery in patients with metastatic prostate cancer include hormone status and Karnofsky performance score [23]. The current study adds to this list by showing that patients with myeloma-like bone metastases and/or low PSA IR scores have significantly reduced postoperative survival periods compared with other patients with MSCC. The myeloma-like radiographic appearance provided independent prognostic information from KPS and other clinical and pathological markers and may thus serve as a novel independent negative prognostic marker in the decision-making process for surgical treatment of MSCC. The myeloma-like appearance was not obviously related to a late stage of the disease since two patients with this appearance were diagnosed in a hormone-naïve stage. Notably these two patients had a much worse prognosis than the other hormone-naïve patients in the cohort and overall patients with myeloma-like metastases at presentation with MSCC had shorter survival times after the initiation of ADT for primary prostate cancer

The prediction of postoperative ambulatory function is a key component when selecting patients with MSCC for surgical intervention. Ambulation prior to surgery is the best prognostic factor for post-treatment outcome, while the severity and duration of neurological symptoms are also strongly related to postoperative function [24]. Several radiographic parameters, including the presence of vertebral compression fractures, grades of spinal cord compression, and spinal instability, have been evaluated but have not been shown to be related to ambulatory function [16], [24], [25]. In the current study, postoperative ambulatory function was significantly reduced in the myeloma-like group.

Several markers related to bone remodelling have been suggested to provide prognostic information related to overall survival in men with bone metastases from CRPC. Low plasma levels of alkaline phosphatase and bone-specific alkaline phosphatase, and low urine levels of N-telopeptide, all showed strong correlations with longer survival times [6]. Only a few cases with myeloma-like prostate cancer bone metastases have been reported in the literature [7], [8], [9], and Idowu [7] has described this metastatic pattern as osteolytic. In our study, most of the patients with MSCC had a mixed osteoblastic and osteolytic or pure osteolytic appearance on CT or MRI, while very few pure osteoblastic cases were observed, and no difference was observed in the amount of osteolysis between the groups with the myeloma-like and non–myeloma-like patterns. The morphological analyses also did not indicate any differences related to bone remodelling between the groups. Thus, it is unlikely that osteolysis is responsible for the aggressiveness of myeloma-like prostate cancer bone metastases.

Some studies have suggested an association between multiple myeloma and prostate cancer. Kao and Jani [26] found an increased incidence of multiple myeloma in their cohort of 700 prostate cancer patients. They highlighted the similarity of the tumour microenvironment for both malignancies during progression. Indeed, there are several case reports with synchronous occurrence of prostate cancer in the bone marrow and multiple myeloma [27], [28], [29], and common genetic variants for multiple myeloma and prostate cancer have been proposed based on the increased risk for multiple myeloma in families with a high incidence of prostate cancer [30]. We did not find any synchronous metastases in our study, and all samples were histologically confirmed as prostate cancer.

### 4.1 Limitations

The main limitation of the study is the relatively small sample size. The bone metastases were prospectively collected, but identification of the myeloma-like prostate cancer bone metastases on MRI was performed retrospectively. The medical and surgical treatments were not randomised, but were rather chosen according to the preference of the surgeon and the oncological teams. Furthermore, the long span of data collection creates another limitation as advances in both diagnostic techniques and adjuvant therapy may influence detection rates of primary tumours, classifications to specific subgroups, and therapy opportunities, and therefore survival.

## 5 Conclusions

A myeloma-like MRI appearance of bone metastases may be present in a substantial proportion of prostate cancer patients with MSCC and is associated with particularly poor survival and neurological function after surgery for MSCC. The biology underlying myeloma-like prostate cancer bone metastases requires further exploration.

  ***Author contributions*:** Johan Wänman had full access to all the data in the study and takes responsibility for the integrity of the data and the accuracy of the data analysis.

  *Study concept and design*: Crnalic, Wikström, Wänman.

*Acquisition of data*: Wänman, Abul-Kasim, Wikström, Bergh.

*Analysis and interpretation of data*: Wänman, Wikström.

*Drafting of the manuscript*: Wänman, Wikström, Crnalic.

*Critical revision of the manuscript for important intellectual content*: Wänman, Abul-Kasim, Semenas, Thysell, Bergh, Wikström, Crnalic.

*Statistical analysis*: Wänman, Crnalic, Wikström.

*Obtaining funding*: Crnalic.

*Administrative, technical, or material support*: Wikström, Bergh, Semenas, Thysell.

*Supervision*: Crnalic, Wikström.

*Other*: None.

  ***Financial disclosures:*** Johan Wänman certifies that all conflicts of interest, including specific financial interests and relationships and affiliations relevant to the subject matter or materials discussed in the manuscript (eg, employment/affiliation, grants or funding, consultancies, honoraria, stock ownership or options, expert testimony, royalties, or patents filed, received, or pending), are the following: Anders Bergh and Pernilla Wikström have a pending patent application (“Methods for diagnosis and prognosis of prostate cancer”, EP2020/054682). All other authors have no conflicts of interest.

  ***Funding/Support and role of the sponsor*:** The Swedish Cancer Society, the Cancer Research Foundation in Northern Sweden, and the County of Västerbotten grant funds were received in support of this work.

  ***Acknowledgements*:** The authors thank Pernilla Andersson and Susanne Gidlund for technical assistance.
